# Targeting the active site of the placental isozyme of alkaline phosphatase by phage-displayed scFv antibodies selected by a specific uncompetitive inhibitor

**DOI:** 10.1186/1472-6750-5-33

**Published:** 2005-12-22

**Authors:** Deepti Saini, Mrinalini Kala, Vishal Jain, Subrata Sinha

**Affiliations:** 1 Department of Biochemistry, All India Institute of Medical Sciences, New Delhi-110029, India; 2 University of Arkansas for Medical Sciences, USA; 3 University of California, San Francisco, USA

## Abstract

**Background:**

The isozymes of alkaline phosphatase, the tissue non-specific, intestinal and placental, have similar properties and a high degree of identity. The placental isozyme (PLAP) is an oncofetal antigen expressed in several malignancies including choriocarcinoma, seminoma and ovarian carcinoma. We had earlier attempted to isolate PLAP-specific scFv from a synthetic human immunoglobulin library but were unable to do so, presumably because of the similarity between the isozymes.

In this work, we have employed a PLAP-specific uncompetitive inhibitor, L-Phe-Gly-Gly, to select isozyme specific scFvs. An uncompetitive inhibitor binds to the enzyme in the presence of substrate and stabilizes the enzyme-substrate complex. Several uncompetitive inhibitors have varying degrees of isozyme specificity for human alkaline phosphatase isozymes. A specific uncompetitive inhibitor would be able to unmask conformational differences between the otherwise very similar molecules. Also, such inhibitors would be directed to regions at/close to the active site of the enzyme. In this work, the library was first incubated with PLAP and the bound clones then eluted by incubation with L-Phe-Gly-Gly along with the substrate, para-nitro phenyl phosphate (pNPP). The scFvs were then studied with regard to the biochemical modulation of their binding, isozyme specificity and effect on enzyme activity.

**Results:**

Of 13 clones studied initially, the binding of 9 was inhibited by L-Phe-Gly-Gly (with pNPP) and 2 clones were inhibited by pNPP alone. Two clones had absolute and 2 clones had partial specificity to PLAP. Two clones were cross-reactive with only one other isozyme. Three scFv clones, having an accessible His6-tag, were purified and studied for their modulation of enzyme activity. All the three scFvs inhibited PLAP activity with the kinetics of competitive inhibition. Cell ELISA could demonstrate binding of the specific scFvs to the cell surface expressed PLAP.

**Conclusion:**

The results demonstrate the biochemical modulation of scFv binding. Also, the scFvs bound to the active site and denied the access to the substrate. The selection strategy could generate specific anti-enzyme antibodies to PLAP that can potentially be used for targeting, for modulating enzyme activity in *in vitro *and *in vivo *and as probes for the active site. This strategy also has a general application in selecting antibodies from combinatorial libraries to closely related molecules and conformations.

## Background

The alkaline phosphatases (APs) are a family of enzymes with a number of isozymes and isoforms that differ from each other in various degrees of amino acid sequences and the extent and nature of glycosylation. In humans, three of the four AP isozymes aretissue specific, i.e., the intestinal AP (IAP), placental AP (PLAP), and germ cell AP (GCAP), while the fourth AP gene is the tissue-nonspecificAP (TNAP) found expressed in bone, liver, and kidney [1]. There is 50% identity between TNAP and PLAP and 86% identity between Intestinal and Placental isozyme at the level of protein sequence [2-5]. In this study, TNAP is represented by bone isozyme (BAP). The postulated functions of the isozymes are many [6-8]. While the ubiquitous expression of AP family across the phyla and also within the human body points to a broad conservation of important functions, the diversity of the isozymes and isoforms also indicates a certain degree of differentiation and specificity regarding their functions.

Our laboratory has been working on the generation of recombinant antibodies to PLAP for possible use in tumor targeting [9,10]. PLAP is expressed on the cell surface in several types of malignancies [11], including choriocarcinomas, seminomas, and tumors of ovary, uterus, cervix, breast, lung, stomach and bladder. Even though the percentage expression in a particular tumor type is variable, the total numbers of tumors expressing the antigen are quite high, and encompass a range of solid tumors. Most of the current management strategies for solid tumors have a poor outcome. Certain characteristics of PLAP, like cell surface localization [12], clathrin mediated endocytosis [13] and low shedding into circulation makes it an ideal target for immunolocalization and immunotherapy [14]. Antibodies specific to PLAP would be useful for localizing therapeutic modalities like conjugated toxins, drugs and liposomes carrying cytotoxic compounds as well as for tumor imaging. In our earlier work [9,10], we had attempted to select phage clones exhibiting isozyme specific binding from a phage-displayed human scFv library [15]. As is usually done, we had selected anti-PLAP scFv by allowing the phage library to bind to immobilized PLAP and eluted with high pH. Though we could select clones that bound the selecting antigen, we failed to isolate PLAP-specific clones. This highlighted the need for adopting alternative strategies for isolating phage antibodies that bind to defined structural regions in an isozyme specific manner.

It would be a rational strategy to use small molecules that bind to defined regions of the selecting antigen to elute out bound phage from specific antigenic conformations. For enzymes in general, the detailed studies on substrates and inhibitors available in literature suggest ways for carrying out such selections. There are several aspects of the catalysis by AP family that offer clues to conformations that could be exploited for the selective targeting of the isozymes. While all of the isozymes can catalyse several small molecular artificial substrates (like pNPP), even within the isozymes expressed in human tissues there are several uncompetitive inhibitors that have varying degrees of isozyme specificity. L-Phe-Gly-Gly and L-Leu are PLAP-specific, while L-Phe inhibits both PLAP and IAP [16]. L-Homoarginine specifically inhibits TNAP [16]. The binding patterns of these inhibitors perhaps reflect homologies or unique structures in these molecules [16,17]. An uncompetitive inhibitor is effective only in the presence of substrate [16]. Uncompetitive inhibitors bind to the enzyme-substrate complex and their binding site would therefore be at/close to the active site of the enzyme [18,19]. It was expected that L-Phe-Gly-Gly (along with pNPP) would; therefore, elute phage bound to the definite structures on PLAP that were unique to the isozyme. Such a strategy would also provide a way for the directed selection of isozyme specific phage antibodies. X-ray crystallography has revealed that the active site of PLAP is a large shallow depression [20]. Therefore, it would be possible for such scFvs to interact directly with the active site and also modulate PLAP function.

In this study, we have incubated the phage-displayed scFv library with PLAP and eluted the phage clones bound to the immobilized PLAP by L-Phe-Gly-Gly in the presence of pNPP. These clones have been studied with regard to their isozyme specificity, biochemical modulation of binding and the effect of their binding on the enzyme activity. This strategy has enabled us to isolate a high proportion of scFv antibodies that are isozyme specific and bind to the active site, as evidenced by the modulation of enzyme activity. The binding characteristics of these phage antibodies provide useful information regarding the conformational relatedness of the AP isozymes and thus could also serve as structural probes.

## Results

### Selection of PLAP binding clones and determination of antigen binding

**Figure 1:**
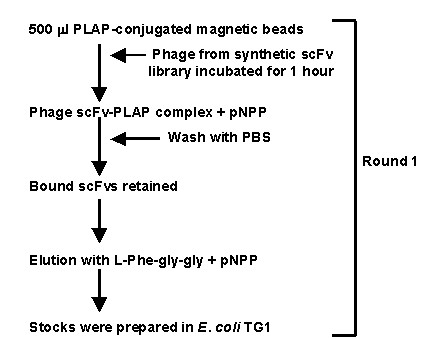
Flow Chart of the selection strategy.

**Figure 2:**
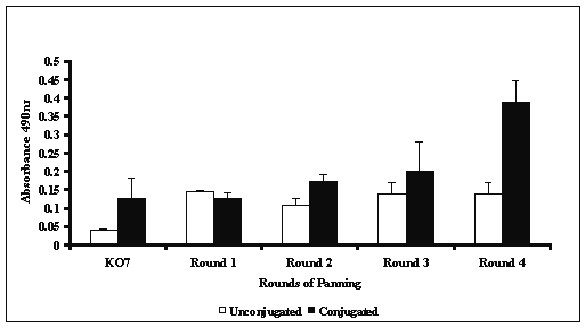
**Polyclonal MBPhE. **10^11 ^pfu of each round phage eluted with L-Phe-Gly-Gly + pNPP were checked for binding affinity to PLAP-conjugated magnetic beads (solid bars) against the background binding of phage to the unconjugated beads (white bars). The black bars on top indicate SD.

The pH of 9.6, which is optimal for PLAP activity, would probably denature the scFv displayed on the phage and also disrupt PLAP-scFv interaction. Hence, after standardization, a pH of 8.0 was chosen for elution with 0.01 M L-Phe-Gly-Gly. At this pH, PLAP activity was retained and the enzyme activity was inhibited by L-Phe-Gly-Gly but not by the control peptide L-Gly-Gly-Gly. Phage eluted by L-Phe-Gly-Gly + pNPP (Figure 1), were amplified after each round of selection and the binding of phage to PLAP was assessed by Magnetic Bead Phage ELISA (MBPhE). The fourth round phage showed significant binding to PLAP (Figure 2). Hence, individual phage clones from the fourth round were assessed for their ability to bind PLAP.

### Enrichment in Transforming units (TUs) of PLAP binding phage after each round of selection

The Transforming Units (TUs) of input and output phage after each round of selection are shown in Table 1. There was an initial fall in the TUs of the eluted phage in comparison to the input phage. Also, we observed a decrease in the TUs of the phage eluted after the second round of selection indicating the decrease in phage binding non-specifically to beads. There was an increase in phage TUs in subsequent rounds of selection indicating enrichment of selected anti-PLAP phage.

### Antigen binding characteristics of monoclonal anti-PLAP scFvs

**Figure 3:**
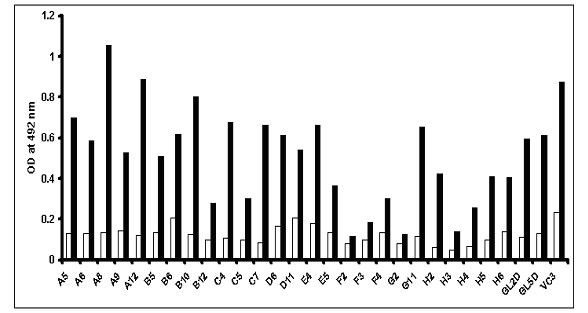
**PLAP binding activity of the 30 phage clones (of the 100 tested) that showed positive binding in the Magnetic Bead Phage ELISA. **The solid bars represent binding of the each phage clone to the PLAP-conjugated magnetic beads. The empty bars represent binding activity of phage clones to the unconjugated magnetic beads (negative control).

100 phage clones from the fourth round of selection were assessed for PLAP binding by MBPhE. This was performed like the polyclonal MBPhE. 30 PLAP binding clones were detected (Figure 3). Out of these, 13 clones were selected on the basis of extent of binding and were further characterized.

### Magnetic Bead Phage ELISA (MBPhE) in the presence of inhibitors

**Figure 4:**
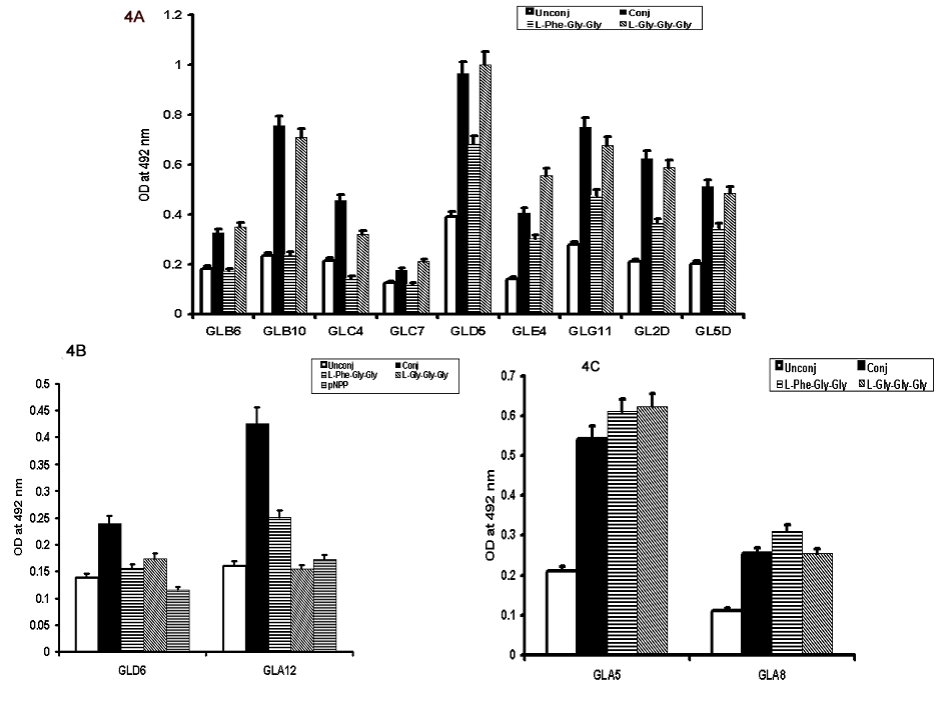
***Binding features of Type I, Type II and Type III antibodies on MBPhE. 4A. ***Representative binding of *Type I *antibodies on MBPhE. The binding of these antibodies was inhibited by L-Phe-Gly-Gly + pNPP but not by L-Gly-Gly-Gly + pNPP. The unconjugated magnetic beads served as a negative control. The black bars on top indicate SD. ***4B. ***Representative binding of *Type II *antibodies on MBPhE. The binding of these antibodies was inhibited both by L-Phe-Gly-Gly + pNPP and L-Gly-Gly-Gly + pNPP and by substrate alone. The unconjugated magnetic beads served as a negative control. The black bars on top indicate SD. ***4C. ***Representative binding of *Type III *antibodies on MBPhE. The binding of these antibodies was not inhibited by L-Phe-Gly-Gly + pNPP or L-Gly-Gly-Gly + pNPP. The unconjugated magnetic beads served as a negative control. The black bars on top indicate SD.

This was done for the 13 clones that gave significant binding to PLAP. Binding was studied in the presence of the inhibitor L-Phe-Gly-Gly or the control peptide L-Gly-Gly-Gly along with the substrate pNPP. Based on the inhibition of antigen binding by substrate and L-Phe-Gly-Gly/L-Gly-Gly-Gly, these clones were of three types. *Type I *(Figure 4A) comprised of the clones whose binding was inhibited by L-Phe-Gly-Gly but not by L-Gly-Gly-Gly (9 clones were of this type including the clones GLE4 and GL2D). This indicated that the bound clones were displaced from PLAP on the formation of the enzyme-substrate-inhibitor complex. *Type II *phage antibodies (Figure 4B) comprised of 2 clones whose binding was inhibited by substrate alone (clones GLD6 and GLA12). The inhibitor, L-Phe-Gly-Gly or the control peptide L-Gly-Gly-Gly did not affect the binding. This suggested that while the clone was displaced on the binding of the substrate PLAP, the inhibitor did not influence the binding. *Type III *phage antibodies (Figure 4C) comprised of 2 clones whose binding was not inhibited by either L-Phe-Gly-Gly or L-Gly-Gly-Gly (clones GLA5 and GLA8). The binding of these clones was not inhibited by either substrate or inhibitor and suggested that their elution was presumably physico-chemical.

### Relative binding to the various isozymes of alkaline phosphatase

**Figure 5:**
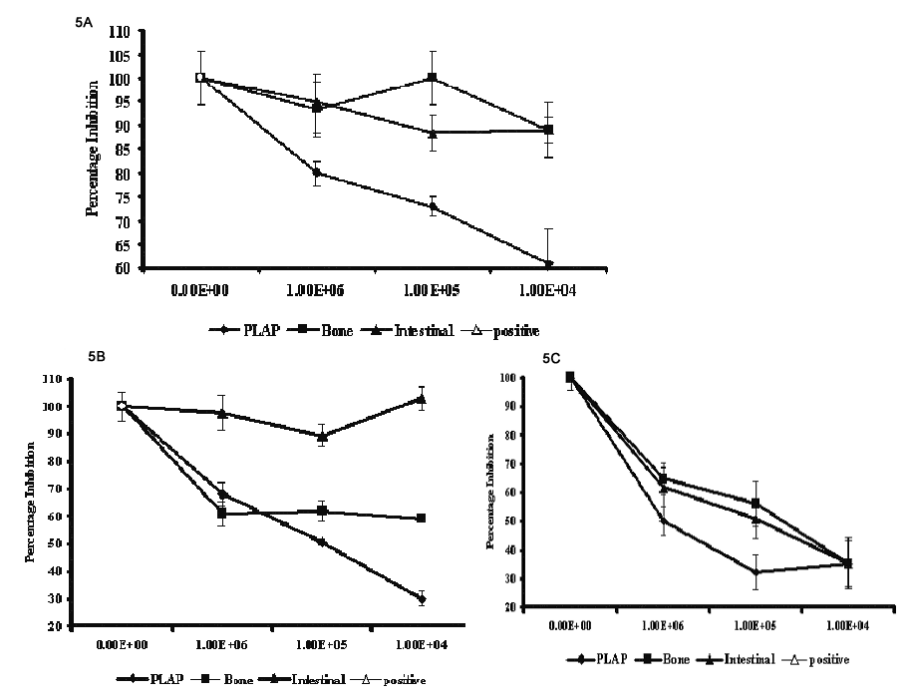
***Characteristics of PLAP-scFv binding in the presence of competing isozymes. 5A. ***A fixed amount of biotinylated-PLAP was incubated with immobilized soluble scFv of the clone GLE4. The extent to which this could be inhibited by varying concentrations of competing isozymes was measured. Binding in the absence of any competing isozyme was taken as 100%. The binding to biotinylated-PLAP is inhibited only by unbiotinylated PLAP and not the other two isozymes of alkaline phosphatase. The labels on x-axis represent the molar concentration of the various isozymes included in the experiment. ***5B. ***Competition of PLAP-GLD6 binding by IAP and BAP. Normalisation of the data was done as in *Figure 6A*. The binding to biotinylated-PLAP is inhibited by the two isozymes (BAP and PLAP) but not the third. The labels on x-axis represent the molar concentration of the various isozymes included in the experiment. ***5C. ***Competition of PLAP-GL2D binding by IAP and BAP. Normalisation of the data was done as in *Figure 6A*. Binding to biotinylated-PLAP binding was inhibited by all the isozymes of alkaline phosphatase. At the maximum concentration of competing isozyme studied, the inhibition was the same. However, there was a slight difference in the relative affinity when 50 % inhibition was calculated. The labels on x-axis represent the molar concentration of the various isozymes included in the experiment.

The binding of scFvs to biotinylated-PLAP was competed with the increasing molar concentrations of unbiotinylated PLAP, IAP or BAP. The binding of the studied clones to biotinylated-PLAP was affected in the presence of unbiotinylated soluble isozymes (PLAP, BAP, IAP) to a different extent, depending on the clone. The binding of two clones (clones GLE4 and GL2D) to biotinylated-PLAP was inhibited only by unbiotinylated PLAP and not the other two isozymes of alkaline phosphatase (Figure 5A). The binding of two clones was inhibited by unbiotinylated PLAP at much lower molar concentrations than by the other two isozymes. This indicated that selection strategy led to the preferential isolation of PLAP-specific scFvs. For 3 clones (including clone GLD6) binding to biotinylated-PLAP was inhibited in the presence of two isozymes (BAP and PLAP) but not the third (Figure 5B). However, seven clones (including clone GLA8) exhibited inhibition of binding to biotinylated-PLAP in the presence of all the three isozymes of alkaline phosphatase (Figure 5C).

### Influence of antibody binding on PLAP/IAP/BAP enzyme activity

**Figure 6:**
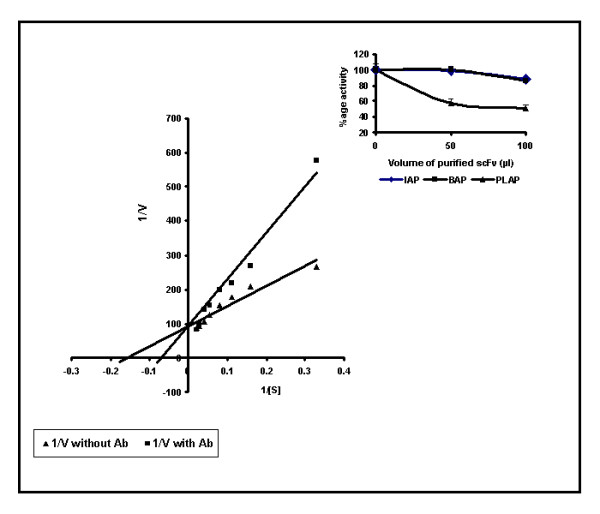
***Lineweaver-Burk Plot of inhibition of PLAP activity by purified GLE4. ***The enzyme activity of PLAP is inhibited in a competitive manner. Km of the enzyme: 5.5 mM; Km of the enzyme with GLE4: 40 mM. Inset shows % inhibition of enzyme activity by varying amount of purified scFv GLE4 (This clone also shows a higher binding [by ELISA] to PLAP as compared to BAP or IAP).

**Figure 7:**
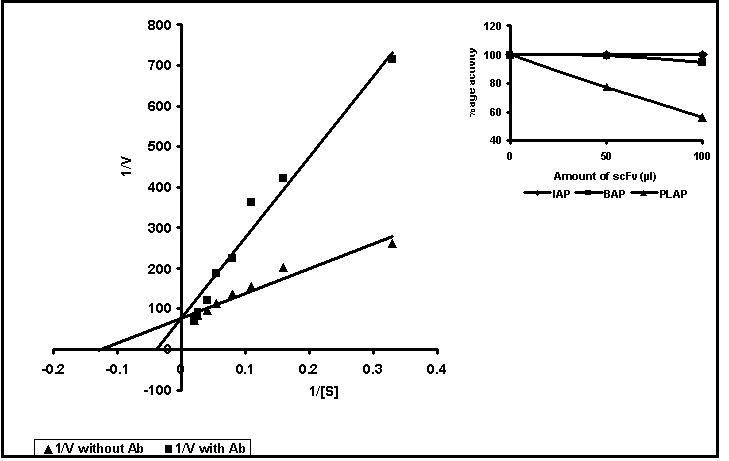
***Lineweaver-Burk Plot of inhibition of PLAP activity by purified GL2D. ***The enzyme activity of PLAP is inhibited in a competitive manner. Km of the enzyme: 5.5 mM; Km of the enzyme with GL2D: 25 mM. Inset shows % inhibition of enzyme activity by varying amount of purified scFv GL2D (This clone also shows a higher binding [by ELISA] to PLAP as compared to BAP or IAP).

**Figure 8:**
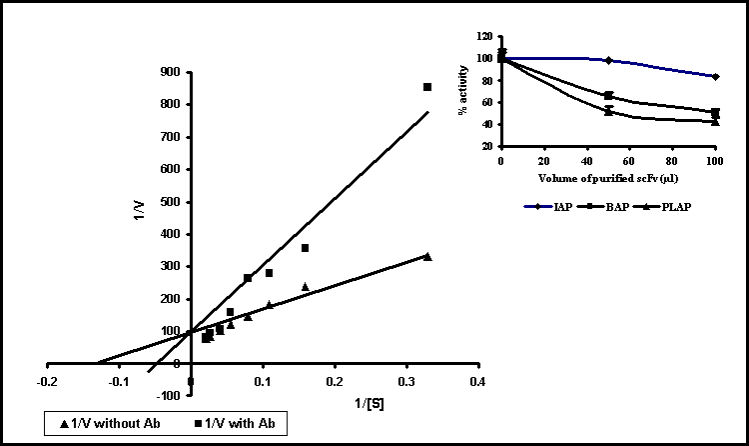
***Lineweaver-Burk Plot of inhibition of PLAP activity by purified GLD6. ***The enzyme activity of PLAP is inhibited in a competitive manner. Km of the enzyme: 5.5 mM; Km of the enzyme with GLD6: 15 mM. Inset shows % inhibition of enzyme activity by varying amount of purified scFv GLD6 (This clone has a higher binding [by ELISA] to PLAP and BAP as compared to IAP).

Three scFv antibodies, GLE4, GLD6 and GL2D, had an accessible His_6_-tag and were purified on metal affinity columns. There is also a possibility that the His-tag was not detectable in the other clones because of their poor expression levels, but this has not been explored further. The ability of the three selected clones to modulate the activity of the three isozymes, PLAP, IAP and BAP was studied in detail. Clones GLE4 and GL2D specifically inhibited the activity of PLAP but not of the other isozymes (IAP and BAP). Clone GLD6, that binds to both PLAP and BAP, inhibited the activity of these two isozymes but not of IAP. The nature of inhibition of PLAP activity by these scFv antibodies was studied using varying substrate concentrations. Lineweaver-Burk (1/V vs. 1/[S]) plots were made for GLE4, GL2D and GLD6 (Figures 6, 7 and 8*respectively*). All the mentioned scFv clones inhibited enzyme activity. The plots in the presence of the scFvs suggested a competitive type of inhibition as the inhibition was completely relieved by higher concentrations of the substrate, indicating competition by the antibody and substrate for a site on the PLAP molecule. For the Clone GLE4, Km of the enzyme was 5.55 mM; Enzyme with scFv: K'm: 11.11 mM and Ki ~10^8 ^M^-1^. For the Clone GLD6, Km of the enzyme was 5.55 mM; Enzyme with scFv: K'm: 15 mM and Ki ~10^9 ^M^-1^. For the Clone GL2D, Km of the enzyme was 5.55 mM; Enzyme with scFv: K'm: 25 mM and Ki ~10^8 ^M^-1^.

### Binding of the purified scFvs to PLAP expressed on tumor cell lines

**Figure 9:**
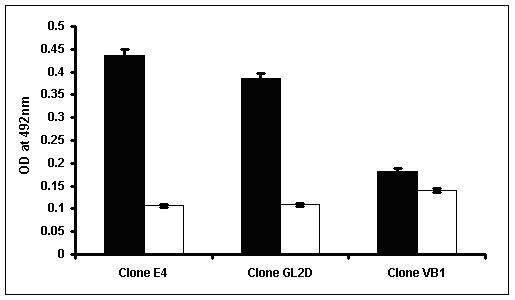
***Cell ELISA to study the binding of the isozyme specific scFvs to the cell surface expressed PLAP. ***The two PLAP-specific purified scFvs, clone E4 and clone GL2D showed significant binding to the HeLa cells that express PLAP (solid bars). SaOs-2 cells, that do not express PLAP, served as control (empty bars). The scFv VB1 did not show binding to either of the cell types and served as a negative scFv control. The black bars on top indicate SD.

The two scFv clones, E4 and GL2D, showed binding to the purified PLAP in an isozyme specific manner. The binding of these scFvs was then studied on HeLa cell line that is known to express PLAP. Cell ELISA could detect binding of these two clones in a PLAP-specific manner. Both the clones showed binding to PLAP expressed on HeLa cells (Figure 9).

### Sequence analysis

Identity analysis of the scFv sequences was performed at NCBI BLAST server. The sequences showed identity to immunoglobulin heavy and light chain variable fragments. The sequences of the selected clones have been submitted to EMBL and their accession numbers are as follows: clone GLE4-AJ704539, clone GL2D- AJ704837 and cloneGLD6-AJ704834. The germline origin of all the sequences were determined using DNAPlot at the VBASE database and the data is shown in Table 2. All the sequences were numbered as per Kabat convention. No domain deletion or frame shifts were observed in these sequences.

## Discussion

Various selection strategies for phage-displayed antibodies have been described, especially for large combinatorial libraries with high degrees of diversity. The selection strategy is very important in determining the binding characteristics of the phage [21]. The implementation of specific biochemical methods of selection, wherever possible, would give the selected phage a certain degree of pre-determined epitope specificity. In the case of human alkaline phosphatases a unique panel of uncompetitive inhibitors permits the distinction between the various isozymes, in spite of their having a high degree of sequence identity. In this work, phage antibodies were eluted with a PLAP-specific uncompetitive inhibitor, L-Phe-Gly-Gly, in the presence of substrate, to select clones that are likely to be isozyme specific. By definition, an uncompetitive inhibitor binds to the enzyme-substrate complex and hence would be likely to bind close to the active site of the enzyme.

Our selection strategy entailed the use of optimal pH for phage viability and enzyme inhibition. We have used alternating surfaces of magnetic beads and plastic immunotubes to avoid selection of phage binding to the solid support. The fourth round polyclonal phage showed significant binding to PLAP. Next, the binding of monoclonal scFvs was studied in the presence of L-Phe-Gly-Gly or L-Gly-Gly-Gly (along with the substrate, pNPP). For most of the clones (*Type I*), binding was inhibited by L-Phe-Gly-Gly + substrate, indicating that they bound to a region of PLAP from where they were displaced by the specific inhibitor. Of the three clone studied in detail (Clones GL2D, GLE4 and GLD6), two (GL2D and GLE4) were of *Type I*. They bound only to PLAP. However, for two clones (GLD6 and GLA12), the substrate (pNPP) alone was sufficient to displace the bound scFv, indicating their binding to the active site of the enzyme. Since the synthetic substrate is common to all the three isozymes, the conformation of the binding site for such clones is likely to be a shared one. One such clone (GLD6) has been studied in detail. This was cross-reactive with BAP but not with IAP. This is interesting because PLAP has a greater overall similarity with IAP than with BAP. It is probable that the displaced scFv made contact with those conformations of PLAP that were shared with BAP but not IAP.

The modulation of enzyme activity by the purified scFvs reflected that the selection strategy was targeted towards the active site of PLAP. All the three clones inhibited the activity of the bound isozyme. In all the cases, the inhibition was completely relieved by excess substrate indicating competition for the same binding site. The Alkaline Phosphatase structure reveals five crucial regions, namely theactive site and its vicinity, the active site valley, the homodimerinterface, the crown domain, and the metal-binding site [20]. The active site is a shallow groove, suggesting that phospho-proteins could be natural substrates for alkaline phosphatase *in vivo*. The modulation of enzyme activity by the scFv would support the notion of an active site accessible to large molecules. The soluble scFv, when it bound to the enzyme, presumably denies the substrate access to the active site. The asymmetry of the molecular weights of pNPP and scFv is also worth noting. The scFv, being a large molecule, cannot form a bridge between the enzyme-substrate complex and the crown and acts as a competitive inhibitor possibly by extruding the substrate from the active site.

The crown domain and the metal-binding domain are mammalian-specific andwere observed for the first time in the PLAP structure. The domain is said to be involved in the properties of the enzyme that distinguish one isozyme form the other [22]. The binding of uncompetitive inhibitors like L-Phe and L-Leu have been studied and modeled extensively in earlier studies [23]. Both inhibit PLAP and IAP. The inhibitor acts by functionally interacting with Arg 166 and Zn^2+ ^ion through its carboxy and amino groups respectively, and the conformation of the unique surface loop located in the vicinity of the active site influences both the positioning of the inhibitor and its accessibility to the active site [24]. Mutations of PLAP at residue 367 with Ala and Phe, as well as the corresponding double mutants containing Gly 429 have been shown to result in a 2-fold decrease in *k*cat, a significant decrease in heat stability, and disruption of inhibition by the uncompetitive inhibitors L-Phe and L-Leu [23]. Because the crystal structure has only been elucidated for the binding of L-Phe to PLAP, such antibody probes would be useful for further exploring the binding of these inhibitors. While the binding of L-Phe-Gly-Gly has not been so extensively studied, the site is probably overlapping but not identical to that of L-Leu. The binding sites of L-Leu and L-Phe-Gly-Gly are probably located close to each other but may not be identical [24].

The present study demonstrates that isozyme specific inhibitors can be used to select phage antibodies that in many cases, bind to the active site and show isozyme specificity. Such molecules can distinguish similar conformations and therefore, possibly act as structural probes. In addition, antibodies that inhibit enzyme activity and bind to the cell surface expressed PLAP, could be employed to study the physiological and pathological significance of PLAP, and for targeting tumour cells. In a more general way, the study highlights how the specificity of small molecules can be utilized to select binding entities directed to the specific molecular regions from large and highly diverse combinatorial libraries.

## Conclusion

The results indicate that a small molecule inhibitor can be used to select phage antibodies directed to the active site of PLAP. The binding of most of the scFvs selected by this process was reduced in the presence of the inhibitor. A significant number of clones had various degrees of isozyme specificity. The three clones studied showed inhibition of enzyme activity, suggesting that they bound to the active site and denied the access to the substrate. This indicated the success of the selection strategy. The specific clones showed binding to HeLa cells (that express PLAP). These scFvs could thus be used as probes for the active site of PLAP and to modulate enzyme activity. This strategy also has a general application in selecting specific antibodies from combinatorial libraries to closely related molecules and conformations.

## Methods

*General reagents *– Acetic acid, Ammonium acetate, Chloroform, Disodium hydrogen phosphate, Glycerol, Glycine, Hydrochloric acid, Isopropyl alcohol, Isoamyl alcohol, Methanol, Potassium acetate, Sulphuric acid, Sodium chloride, (AR grade) – *SRL, India or Qualigens, India. *8-Hydroxy quinoline, Acryl amide, Ammonium persulphate (APS), Ampicillin, Bis – Acryl amide, Dextran sulphate, Disodium ethylene diamine tetra acetate (EDTA), Isopropyl thio-β-D-galacto pyranoside (IPTG), Kanamycin, Lysozyme, Magnesium chloride, N,N,N,N'-tetramethylethylenediamine (TEMED), Phenol, Sodium lauryl sulphate (SDS), – *Sigma Chemical Co., St. Louis, USA *or *US Biochemicals, USA *or *SRL, India *or *BDH, England*. Media components – Bacto – agar, Yeast extract, Tryptone, Tissue – culture media; sodium bicarbonate; trypsin – *Sigma Chemicals Co., USA*. Amino acids and proteins – L-Leucine, L-Phe, L-Phe-Gly-Gly, L-Gly-Gly-Gly, L-Homoarginine and Placental alkaline phosphatase, Bone alkaline phosphatase, Intestinal alkaline phosphatase – *Sigma Chemicals Co., USA *and *Calzyme Inc., USA*. Enzymes – Taq DNA polymerase, *SfiI *and *NotI *– Bangalore *Genei, India*. Blotting Membranes And Filters – Nitrocellulose (.45 μm) and 3 M blotting sheets – *Schleicher and Schull, Germany *and *Whatman, USA*. Kits And Systems-Biotinylation kit, *Sigma Chemicals Co., USA*; Bacterial Strains – *E. coli *HB2151 from *MRC, Cambridge/Pharmacia, USA*; *E. coli *XL-1 blue MRF' from *Stratagene, USA*. Cell lines – HeLa cell lines from lab collection.

### Description of the library

The Griffin.1 library is a 'scFv' phagemid library made from synthetic V-gene segments. The library was constructed by re-cloning the heavy and light chain variable regions from the lox library vectors into the phagemid vector pHEN2 [15].

### Phage rescue, precipitation and titration

The phage propagation steps were performed as described in *Kala et al *[9].

### Immobilization of PLAP antigen

PLAP (Sigma Chemical Co., USA) was covalently conjugated to carboxy-terminal magnetic beads using 1-ethyl-3-(3-diethylaminopropyl) carbodiimide (EDCI) as the coupling agent according to the manufacturer's protocol and percentage coupling was determined on the basis of absorption of pre and post coupling PLAP solution. These PLAP-conjugated beads were used for selection of PLAP binding phage from the synthetic immunoglobulin library.

### Panning strategy

Figure 1 summarizes the methodology employed for panning. We observed that incubation of PLAP with 0.01 M L-Phe-Gly-Gly along with 4 mg/ml pNPP substrate at pH 8.0 resulted in inhibition of enzyme activity without affecting phage viability and scFv binding. Hence these conditions were used during panning and isolation of phage clones.

500 μl of PLAP conjugated beads (~100 μg PLAP) and 500 μl unconjugated beads were blocked with 2 % MPBS (non-fat milk PBS) for 2 hours at 37°C. After washing the unconjugated beads with PBS, these were incubated with 1 ml phage (TU ~10^13^) in 2 % MPBS for 30 min on a rocker to reduce non-specific binding. The unbound phage were added to the blocked PLAP- conjugated beads, incubated for 30 minutes on a rotating turntable and then kept static at room temperature (RT) for 90 minutes. After removing unbound phage, the beads were washed 10 times with PBS-Tween (0.05 %) (PBST) followed by 10 washes with PBS. Bound phage were eluted with 1 ml of the substrate, pNPP (4 mg/ml in PBS), for 30 min at RT. This was done in order to elute phage that were directly displaced by the substrate alone. As the substrate was common to all the isozymes, such phage would be more likely to bind to conformations common to all isozymes. After removing these eluted phage, further elution was done with 1 ml of 0.01 M L-Phe-Gly-Gly (inhibitor peptide) solution in PBS containing 4 mg/ml pNPP. The eluted phage were infected into exponentially growing culture of *E. coli *TG1 and plated on large agar/amp dishes for overnight at 37°C. Stocks of selected phage were made by scraping the dishes with 15 % glycerol in 2xTY. Phage from these stocks were amplified for further rounds of selection. In the second round of panning, the volume of PLAP conjugated beads were reduced to 250 μl. and the initial substrate (pNPP) elution step was not performed. Elution was only by a combination of L-Phe-Gly-Gly and pNPP. In the third round of panning the immobilizing surface was changed to immunotubes (Nunc), coated with 400 μl/ml of PLAP in HCO_3_^- ^buffer pH 9.5. This was done to select against the phage binding non-specifically to the immobilizing surface. Elution was first done with pNPP alone and then L-Phe-Gly-Gly + pNPP. The fourth round was similar to the third except that the immunotubes were washed for 15 times with PBST and 15 times with PBS. This stringent washing was a further precaution against non-specific binding.

Stocks of polyclonal phage in *E. coli *TG1 were made after each round of selection and Transforming Units (TUs) were calculated for phage obtained after each elution to evaluate enrichment of the PLAP binders. For monoclonal phage, individual colonies obtained after the fourth round were rescued and precipitated as described earlier.

### Detection of anti-PLAP polyclonal and monoclonal phage-displayed antibodies

#### i) Magnetic Bead Phage ELISA (MBPhE)

This was done essentially as described by *Kala et al *[9]. 10^11 ^TUs of either polyclonal or monoclonal phage were incubated with 100 μl of PLAP conjugated beads (~15–17.5 μg PLAP/100 μl beads). Binding was detected using anti-M13 antibodies followed by anti-mouse-HRPO conjugate (Sigma USA).

#### ii) Inhibition of phage binding to PLAP by substrate and biochemical inhibitors

Uncompetitive inhibitor L-Phe-Gly-Gly or the negative control L-Gly-Gly-Gly were added at the binding step of the MBPhE at a final concentration of 0.01 M along with substrate pNPP (final concentration of 4 mg/ml).

### Sequencing of individual scFv clones

Individual scFv antibodies were analyzed for the presence of scFv DNA insert by *Not *I- *Sfi *I digestion of the phagemid DNA, PCR analysis and subsequent DNA sequencing of the scFv insert. The sequencing primers were pHEN-SEQ (5'CTATGCGGCCCCATTCA 3') and LMB3 (5' CAGGAAACAGCTATGAC 3').

### Expression of phage – displayed antibodies as soluble scFvs

The individual anti-PLAP monoclonal phage displayed antibodies were infected into the non-suppressor strain of E.*coli *HB2151 (OD_600 _~0.5), and after IPTG induction, the periplasmic fraction, containing the soluble scFv, was used for further experiments. This was essentially done as described in *Kala et al *[10].

### Inhibition of PLAP-scFv binding by alkaline phosphatase isozymes

This was done in order to study the competition for the PLAP binding of a scFv clone by equimolar concentrations of the different alkaline phosphate isozymes (BAP and IAP, Calzyme Laboratories Inc.) in comparison to the inhibition resulting from PLAP itself. Firstly, the molar concentration of biotinylated-PLAP giving 50 % binding to scFv was determined. Next, this concentration of biotinylated-PLAP for each scFv clone was used for the competition with soluble PLAP/IAP/BAP. The inhibition of the binding of biotinylated-PLAP by different molar concentrations of BAP, IAP and PLAP was studied by including them at the binding step of the ELISA.

### Purification of scFv clones by metal affinity chromatography

Since the scFv clones expressed as a fusion protein with His_6 _tag at the C – terminal, those having an accessible tag were purified on a metal affinity column (Clontech Inc., USA) as per the manufacturer's instructions. The eluted scFv was dialyzed against 50 mM Tris-NaCl buffer overnight and resolved on a 15 % SDS-PAGE for the analysis of its purity.

### Influence of antibody binding on PLAP/IAP/BAP activity using pNPP as a substrate

This was done in order to determine if the binding antibody is a modulator of the enzyme activity. The effect on the activity of PLAP, IAP and BAP was compared. This was essentially performed in the following described steps.

#### Inhibition assay

0.01 μg of each of the isozymes was incubated with the increasing amount of purified scFv antibody (0–100 μl) and substrate pNPP (100 μl of the stock 4 mg/ml dissolved in Tris buffer, pH 9.0) for 1 hour at RT. The absorbance was read at 405 nm. Percentage inhibition of the activity of each of the isozymes by purified scFv antibodies was plotted.

In the next experiment, the nature of inhibition of PLAP activity by purified scFv antibodies was studied. The concentration of purified scFv antibodies exhibiting 50 % inhibition of the enzyme activity was incubated with increasing substrate concentrations (100 μl of the stock 1 mg/ml to 16 mg/ml, dissolved in Tris buffer, pH 9.0) for 15 minutes at 37°C and pH 9.0. The absorbance was read at 405 nm and 1/V vs. 1/[S] curve was plotted. Also, the calculation of Ki for each of the mentioned clones was done using the following equation for tight-binding inhibitors:

K'm = Km {1 + [I]/Ki} [25]

### Cell ELISA to study binding of scFvs to the cell surface expressed PLAP

HeLa is a cervical carcinoma cell line known to express PLAP. SaOs-2 is an osteosarcoma cell line that does not express PLAP and is therefore, used as a negative control. Tumor cell lines (HeLa and SaOs-2) were cultured in complete medium in 96-well flat bottom plates (Corning) to form a subconfluent monolayer and further incubated overnight in DMEM medium containing 10% FBS. The cells were washed 3 times for 5 min each with phosphate buffered saline (PBS, pH 7.4) and cells were fixed by treatment with 4% paraformaldehyde for 10 min at 4°C. The endogenous peroxidase activity was blocked with 3% H_2_O_2 _in distilled water for 7 min at room temperature followed by 3 × 5 min washes in PBS, pH 7.4. Non-specific binding was blocked with 3% bovine serum albumin (BSA) for 1 h at room temperature. Cells were washed once in PBS and incubated with purified scFvs (E4, GL2D and VB1) diluted in 3%BSA for 2 h at room temperature. After 3 × 10 min washes in PBS, mouse anti-His_6 _IgG antibody (diluted 1/1000 in 1% BSA-PBS) was added for 1 hour at room temperature. The cells were washed again in PBS. Following this, anti-mouse IgG conjugated to HRPO was added and incubated for 1 hour and the bound enzyme activity was developed by adding OPD (5 mg/10 ml citrate buffer, pH 5.0) containing 5 μl of 30% H_2_O_2_. The color reaction was stopped by adding 50 μl of 10% H_2_SO_4 _and the intensity was read at 492 nm in a MicroELISA reader.

## The abbreviations used are

AP, alkaline phosphatase; BAP, bone alkaline phosphatase; DAB, di-aminobenzidine; EDCI, 1-ethyl-3-3-(-3-diethylamonopropyl) carbodiimide; IAP, intestinal alkaline phosphatase; IPTG, isopropylthiogalactosidase; mAb, monoclonal antibody; MBPhE, Magnetic Bead Phage ELISA; MPBS, milk in phosphate buffer saline; OPD, ortho-phenylenediamine; PBS, phosphate buffer saline; PLAP-placental alkaline phosphatase; pNPP, para nitrophenolphosphate; RT, room temperature; SD, Standard Deviation ; TNAP-tissue non specific alkaline phosphatase.

## Authors' contributions

DS, MK and VJ were involved in the selection, purification and characterization of the scFv clones regarding their immunological and biochemical properties. They discussed the data and strategy on a regular basis and were involved in writing the manuscript. SS developed the concept, supervised the project and was involved in writing the manuscript.

## Figures and Tables

**Table 1:** Transforming units (TUs) of PLAP binding phage. Transforming units (TUs) of PLAP binding phage after each round of selection. There was an initial fall in the TUs of the eluted phage in comparison to the input phage. There was also a decrease in the TUs of the phage eluted after the second round of selection indicating the decrease in phage binding non-specifically to the beads. For the rounds one and two, the selection was carried out on magnetic beads. For the rounds three and four, the selection was carried out on immunotubes.

**Rounds of selection**	**Transforming units (input)**	**Transforming units eluted with pNPP (output)**	**Transforming units eluted with L-Phe-gly-gly + pNPP (output)**
Unselected library	10^14^	-	-
First	10^14^	10^2^	10^12^
Second (PLAP- conjugated beads were reduced)	10^14^	THIS STEP WAS NOT PERFORMED FOR THE SECOND ROUND OF SELECTION	10^9^
Third (selection on Immunotubes)	10^14^	10^6^	10^4^
Fourth (selection on Immunotubes)	10^14^	10^7^	10^6^

**Table 2:** Germline origin of all the sequences.

**Clone**			**Germline V sequences**
	
	**EMBL Accession number**	**Heavy Chain (VH)**	**Light Chain (VL)**
Clone GLE4	AJ704539	1-02 (Z14071)	L11 (X93621)
Clone GL2D	AJ704837	**	7a (Z73659)
Clone GLB10	AJ704836	4-30.2 (Z12364)	1e (Z22193)
Clone GL5D	AJ704835	4-59 (Z12371)	9a (Z73675)
Clone GL D6	AJ704834	1-12 (M99638)	1a (Z73653)
Clone GL B6	AJ704538	6-01 (Z12374)	O11/O1 (X93631)
Clone GL A5	AJ704537	1-02 (Z14071)	1g (Z73663)
Clone GL C4	AJ704536	1-02 (Z14071)	1e (Z73656)
Clone GL D5	AJ704535	4-b (M12071)	L12 (X72813)
Clone GL G11	AJ704532	4-30.4 (Z14074)	1g (Z73663)
Clone GL A8	AJ704533	1-02 (Z14071)	1g (Z73663)
Clone GL A12	AJ704534	4-61 (Z12366)	L12 (X72813)

**Insert size of the clone GL2D is 750 bp and there are segments missing in the heavy chain, making it difficult to attribute a matching germline sequence. However, the remaining portions of the heavy chain confirm its identity as part of heavy chain.
